# Neuropathological Changes in the Brains of Suicide Killers

**DOI:** 10.3390/biom11111674

**Published:** 2021-11-11

**Authors:** Tomasz Stępień, Janusz Heitzman, Teresa Wierzba-Bobrowicz, Paweł Gosek, Paweł Krajewski, Agnieszka Chrzczonowicz-Stępień, Jarosław Berent, Tomasz Jurek, Filip Bolechała

**Affiliations:** 1 Department of Neuropathology, Institute of Psychiatry and Neurology, 02-957 Warsaw, Poland; bobrow@ipin.edu.pl; 2 Department of Forensic Psychiatry, Institute of Psychiatry and Neurology, 02-957 Warsaw, Poland; heitzman@ipin.edu.pl (J.H.); pgosek@ipin.edu.pl (P.G.); 3 Forensic Medicine Department, Medical University of Warsaw, 02-007 Warsaw, Poland; krajewskipa@gmail.com; 4 Department of Psychology, SWPS University of Social Sciences and Humanities, 03-815 Warsaw, Poland; achrzczonowicz-stepien@swps.edu.pl; 5 Department of Forensic Medicine, Medical University of Lodz, 91-304 Lodz, Poland; jaroslaw.berent@umed.lodz.pl; 6 Department of Forensic Medicine, Wroclaw Medical University, 50-372 Wroclaw, Poland; tomasz.jurek@umed.wroc.pl; 7 Department of Forensic Medicine, Jagiellonian University Collegium Medicum, 31-531 Cracow, Poland; filip.bolechala@uj.edu.pl

**Keywords:** homicide, suicide, murderer’s brain, murder–suicide, extended suicide, neuropathological changes, neurodegenerative changes

## Abstract

Background: Homicide combined with subsequent suicide of the perpetrator is a particular form of interpersonal violence and, at the same time, a manifestation of extreme aggression directed against oneself. Despite the relatively well-described individual acts of homicide and suicide, both in terms of psychopathology and law, acts of homicide and subsequent suicide committed by the same person are not well-studied phenomena. The importance of emotional factors, including the influence of mental state deviations (psychopathology), on this phenomenon, is discussed in the literature, but still there is relatively little data with which to attempt neuropathological assessments of the brains of suicide killers. This paper is dedicated to the issue based on the neuropathological studies performed. Methods: We analyzed a group of murder–suicides using histochemical and immunohistochemical methods. Results: The results of our research indicate the presence of neurodegenerative changes including multiple deposits of ß-amyloid in the form of senile/amyloid plaques and perivascular diffuse plaques. Conclusions: Neurodegenerative changes found in the analyzed brains of suicide killers may provide an interesting starting point for a number of analyses. The presence of neurodegenerative changes at such a young age in some murderers may suggest preclinical lesions that affect cognitive functions and are associated with depressed moods.

## 1. Introduction

A homicide followed by suicide by the perpetrator is most commonly referred to in the literature as a “murder–suicide” or an “extended suicide”. The number of such cases has remained stable over time and across populations, ranging from 0.05 to 0.3 per populations of 100,000. It is a phenomenon that is classified both as suicide and as homicide, however, it also has separate, distinguishing characteristics that are different from such acts committed individually. As studies show, the perpetrators of murder–suicide are men in 80–90% of cases, and the victims are women (70–80%) and children [1,2,3]. In order not to introduce terminological ambiguity, we have assumed that the term “murder–suicide” used in this paper refers to a particular type of homicide that occurs as a result of a deliberate and planned action. The term “murder–suicide”, in legal terms, would allow for the possibility of an unintentional homicide, as well as the consideration of prosecuting the perpetrator in a criminal trial, which is not usually the case here [4,5]. Among the characteristic types of murder–suicide are: those that are causally related to the disclosure of (1) anger and paranoia; (2) fear of detection; (3) an act of terrorism (suicide bombers), in which suicide is, as it were, “incidental” to the crime [6]. There is relatively little data on the extent to which psychopathological and neurobiological factors, specifically the ones related to organic brain damage in perpetrators, remain associated with impaired emotion control, inhibition, and impulse control. Isolated studies only indicate that among murderers and perpetrators of attempted murders diagnosed with Acute Stress Disorder (ASD) or Posttraumatic Stress Disorder (PTSD) during the act committed, approximately 75% were much more often diagnosed in neuroimaging examinations with frontal lobe atrophy, rather than with temporal lobe atrophy. It was also observed that the ratio of subcortical to cortical atrophy is twice as high, which may be related to the non-accidentally close proximity to the limbic system, which is essential for initiating neurobiological responses to stress [7]. There is insufficient research to support the importance of chronic stress response and neurohormonal, catecholaminergic, inflammatory, and immunosuppressive consequences in motivation and decision-making processes among suicide killers [8]. It is estimated that approximately 25–65% of those who committed murder–suicide had been previously diagnosed with some mental disorder and in most cases this was depression [9]. Some researchers suggest depression with psychotic symptoms [10]. Due to the above characteristics, it is indicated [11] that perpetrators of murder–suicide are more similar to those who commit suicide. However, other authors [12] divide the perpetrators of these acts into two groups: one group with a diagnosis of depression (closer to people who commit suicide) and another group with a history of domestic violence and substance abuse (and therefore closer to perpetrators of murders). Hillebrand [8] points to the possible predominance of biological factors in the aetiology of such events. He refers to the “serotonin-aggression” hypothesis [13], according to which decreased activity of the serotonergic system releases the inhibition of aggression. Examinations of the brains of individuals with a history of aggressive and antisocial behaviour allowed neuroanatomical changes throughout the brain to be described, included amongst which were decreased prefrontal cortex, temporal lobe, and amygdala, and an increased volume of putamen in basal ganglia. An increase in the volume of putamen was attributed to neurodevelopmental disorders, with implications for behavioural disorders, positing that the putamen is involved in an inhibitory mechanism [14]. In addition, a study by Laakso and colleagues demonstrated a negative correlation between antisocial traits and the volume of prefrontal cortex, hippocampus, and amygdala, as well as an increase in impulsiveness and predisposition to aggressive behavior [15]. Significantly smaller volumes of white matter in the frontal lobe in patients diagnosed with schizophrenia-like psychosis, compared to healthy individuals, may be associated with the features of demyelination described in neuropathological studies and a reduced number of oligodendrocytes responsible, among others, for regulating glutamate concentration [16]. For this study we have conducted an analysis of the literature and drawn on our clinical experience and observation of the behaviour of people with behavioural disorders (experience of psychiatric experts). Initiating our study, we asked ourselves whether there were neuropathological changes in the central nervous system in suicidal murderers that could be linked to endo- and/or exogenous factors influencing behaviour at the time of the incident.

## 2. Materials and Methods

The study material consisted of the brains of individuals who had committed murder and subsequent suicide. The materials were taken during autopsies 24–72 h after death (Table 1). All brains were fixed in buffered 4% formaldehyde. Brain autopsies were performed in the Department of Neuropathology, Institute of Psychiatry and Neurology in Warsaw, Poland. Four perpetrators committed suicide by hanging, two by jumping from a height and two by mutilating themselves. All subjects were male. The control material consisted of the brains of men of comparable age, without diagnosed mental illness, whose death occurred as a result of sudden cardiac arrest during a traffic accident with no significant neuropathological changes found. The materials were taken during autopsies 72–96 h after death (Table 2). All brains were fixed in buffered 4% formaldehyde.

The material paraffin embedded was analysed by light microscopy consisted of fragments of the right cerebral hemispheres of eight men aged 19 to 60 (mean 41.13 ± 14.54) in the study group and eight men aged 21 to 80 (mean 42 ± 18.74) in the control group. The analysed material came from the frontal, parietal, occipital, and temporal lobes with the Brodmann area 28, basal ganglia, midbrain, and, in some cases, from the mammillary body and cerebellum. Histological staining was performed with hematoxylin and eosin, cresyl violet, and with modified Bielschowsky’s staining method. Immunohistochemistry (IHC) was also performed with the following antibodies: calbindin D-28k (CaBP) (Sigma, Dramstadt, Germany) at a dilution of 1:2500, GFAP (Bio-Rad, Winninglaan, Belgium) at a dilution of 1:100, GLT-1 (EAAT2, glutamate transporter of glial origin) (LifeSpan, Seattle, USA) at a dilution of 1:100, GLAST (EAAT1, glutamate transporter of neuronal origin) (Bioss, Massachusetts, USA) at a dilution of 1:250, 5-HT_2A_ serotonin (SIGMA, Dramstadt, Germany) at a dilution of 1:100, β-amyloid(1–42) (Bio-Rad, Winninglaan, Belgium) at a dilution of 1:250, β-amyloid(1–40), (Bio-Rad, Winninglaan, Belgium) at a dilution of 1:250.

The study protocol was approved by the Bioethical Commission of the Institute of Psychiatry and Neurology (number 1/2009). The publication was supported by the Institute of Psychiatry and Neurology statutory fund No. 501-026-12014

## 3. Results

### Histochemical Methods

**Figure 1:**
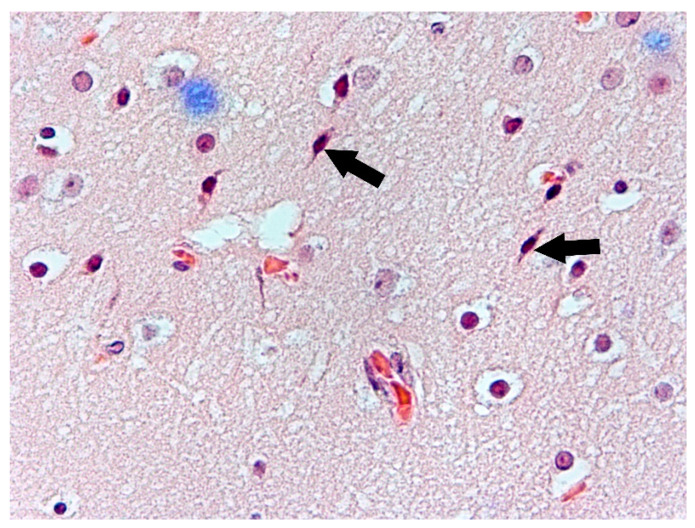
Case no. 4. Visible occipital lobe neurons (arrows) with nuclear hyperchromasia and pyknosis consistent with chronic ischaemic changes. H&E staining, magnification 400×.

**Figure 2:**
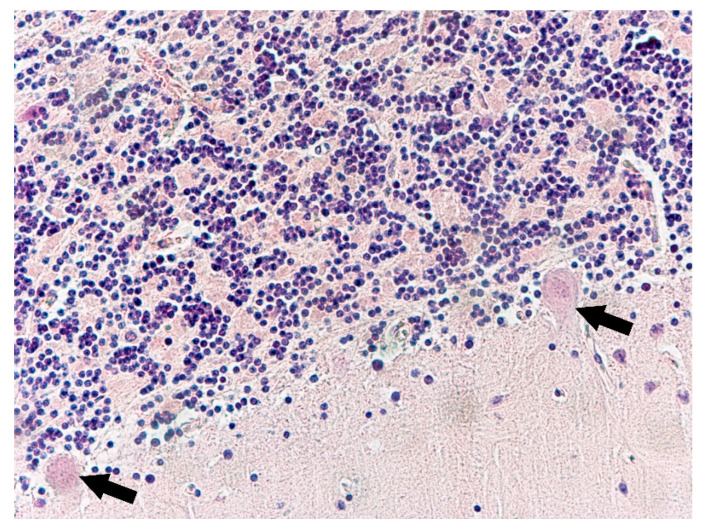
Case no. 1. Cerebellar cortex. Defects and homogenisation changes of Purkinje cells (arrows), Purkinje cells layer. Slight proliferation of Bergmann glial cells and loss of Purkinje cells. H&E staining, magnification 200×.

**Figure 3:**
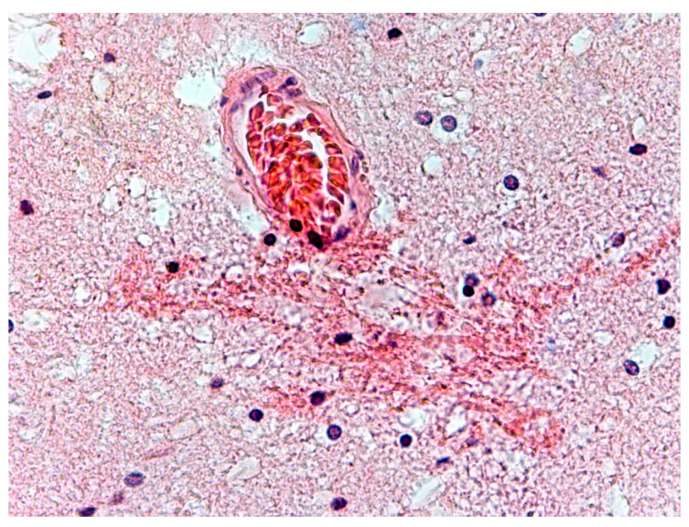
Case no. 4 Mammillary bodies. Damage to the vessel walls. Microbleeds/petechial hemorrhages around the vessels. H&E staining, magnification 400×.

**Figure 4:**
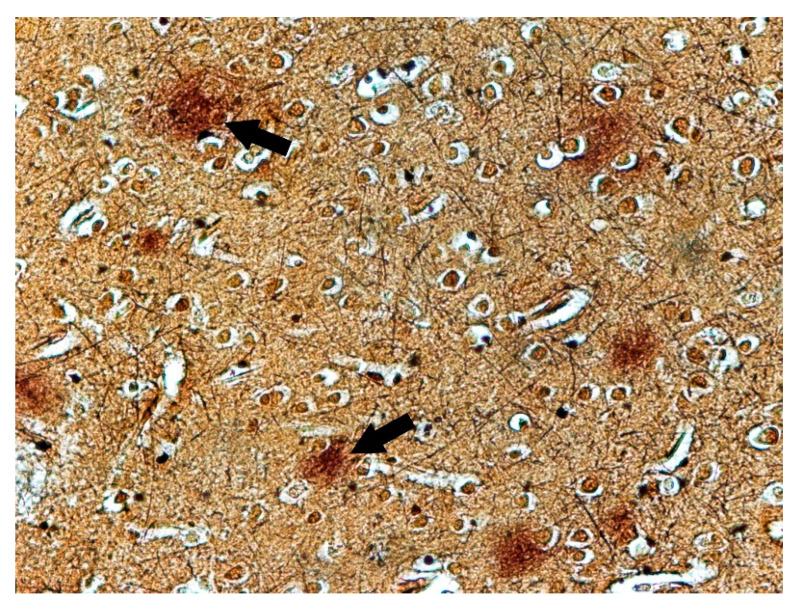
Case no. 1. Temporal lobe of the cortex. Visible senile plaques (arrows). Modified Bielschowsky’s stain, magnification 400×.

**Figure 5:**
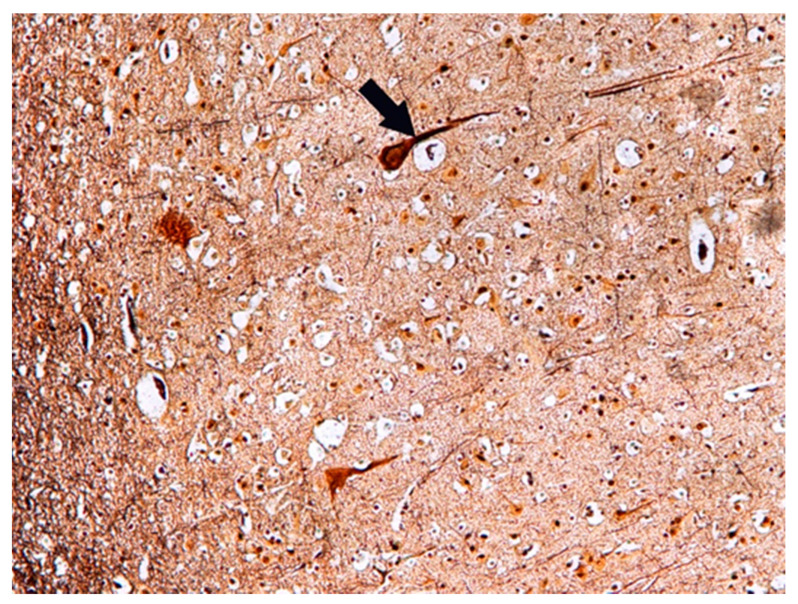
Case no. 8. Frontal lobe of the cortex. Visible neurofibrillary degeneration by silver impregnation with Modified Bielschowsky’s (arrow) stain. Neuronal shrinkage, angularity of cytoplasm and pyknotic nucleus. Magnification 400×.

The changes observed in neurons of the frontal lobe, temporal lobe, occipital lobe, and basal ganglia had the characteristics of chronic lesions/disease (shrinkage of cell body and nucleus, tortuous course of both dendrites and axon), and ischaemic lesions/disease (vivid red in H&E staining) (Figure 1). Changes observed in the cerebellum involved mainly Purkinje cells and cerebellar granule cells. Purkinje cells displayed the characteristics of homogenisation, in which the cytoplasm became homogeneous due to the dispersion of the Nissl substance. This condition was often accompanied by Bergmann glial proliferation (Figure 2). In one case, perivascular petechial haemorrhages were observed in mammillary bodies (Figure 3). Neuroglia proliferation and swelling were observed in the frontal, temporal, and occipital lobes. In four cases the modified Bielschowsky’s staining method revealed the presence of senile plaques (Figure 4) and lesions with the structure of neurofibrillary degeneration (Figure 5). This section may be divided by subheadings. It should provide a concise and precise description of the experimental results, their interpretation, as well as the experimental conclusions that can be drawn.

**Figure 6:**
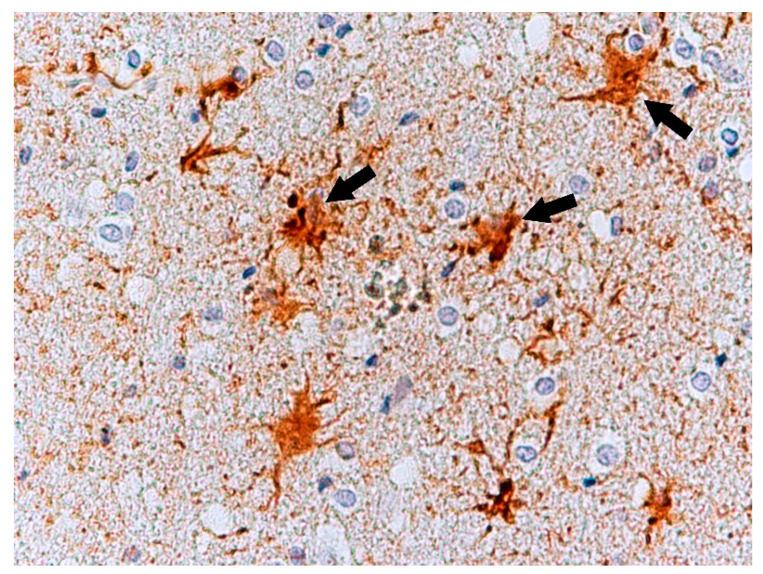
Case no. 3. Frontal lobe of the cortex. Neuropathological changes, damage and fragmentation of astrocytes processes (arrows, clasmatodendrosis). Positive immunohistochemistry reaction with GFAP. Magnification 400×.

**Figure 7:**
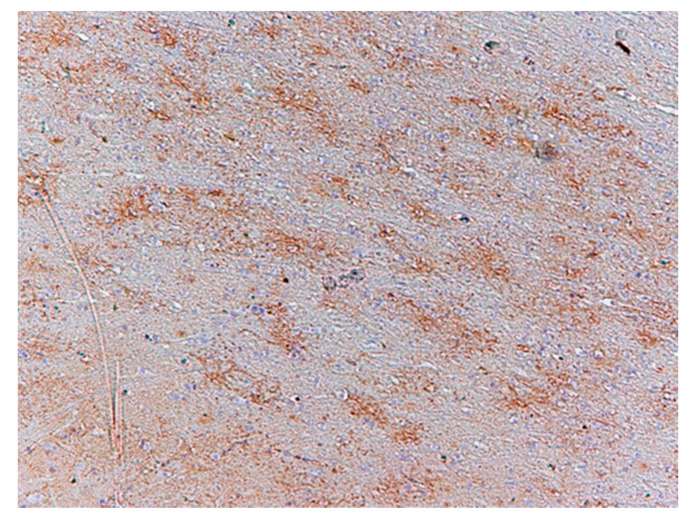
Case no. 8 White matter of the occipital lobe. Positive immunohistochemical reaction with GLT-1. We did not observe any neuropathological changes in glutamate transporter in astrocyte processes. Magnification 200×.

**Figure 8:**
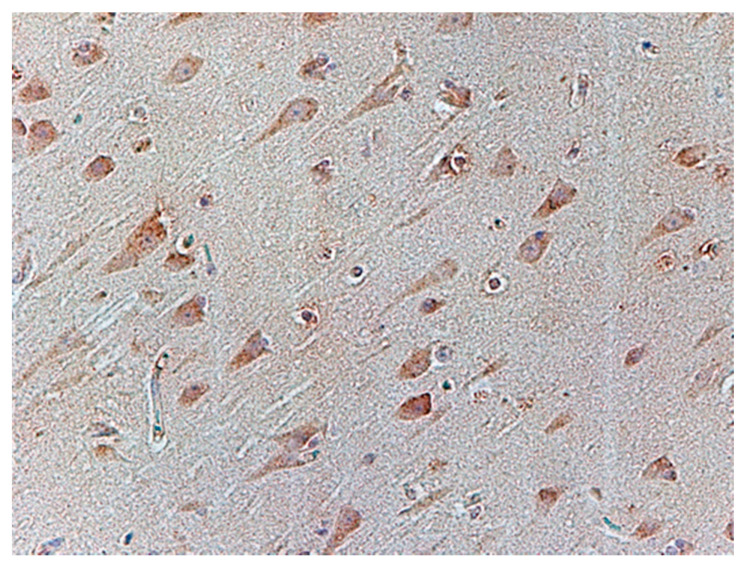
Case no. 3 Frontal lobe of the cortex. Visibly positive immunohistochemical reaction with GLAST in neuronal origin. We did not observe any neuropathological changes in the glutamate transporter in neurons. Magnification 200×.

Immunochemistry for Glial Fibrillary Acidic Protein (GFAP) was positive in astroglia in all the cases studied (Figure 6). Astrocytes were slightly swollen, and their distal processes fragmented (clasmatodendrosis). Disintegration of processes occurred in all immunohistochemically visualized astroglia cells. Antibody staining for glutamate transporters showed an immunohistochemical reaction to GLT-1 in glial cell processes comparable to the control group (Figure 7) and IHC reaction with GLAST in the cytoplasm of neural cells (Figure 8) in the test cases. In contrast, the expression of GLT-1 and GLAST was not observed in two of the studied cases (Table 3).

**Figure 9:**
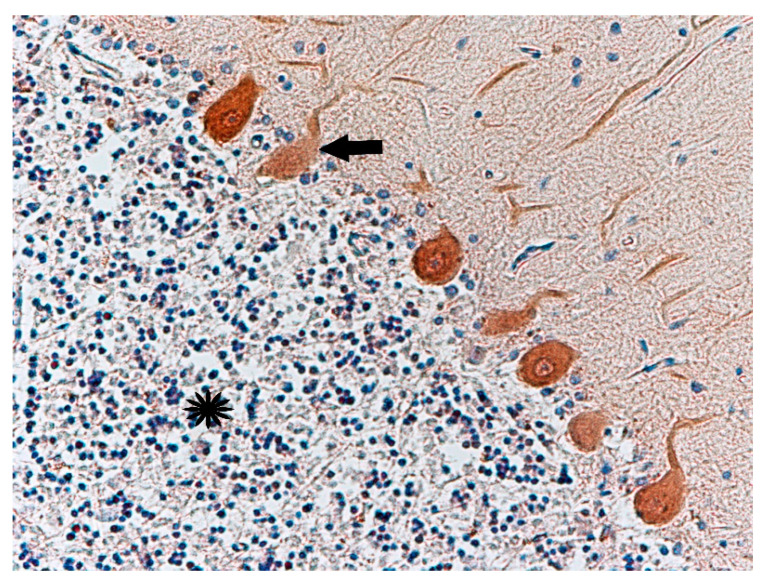
Case no. 2 Cerebellar cortex. Slightly attenuated immunohistochemical reaction with calbindin-D-28k antibody in Purkinje cells (arrow). Slightly thinned neurons in the granular layer (asterisk). Magnification 200×.

**Figure 10:**
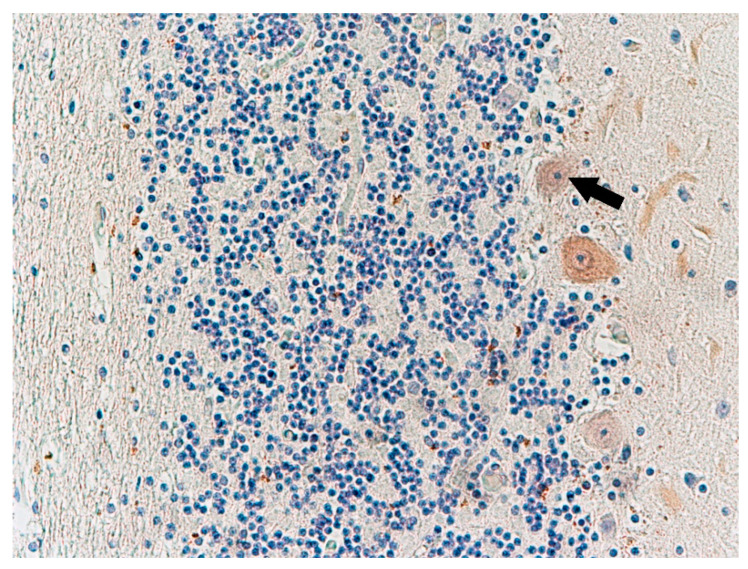
Case no. 1 Cerebellar cortex. Attenuated immunohistochemical reaction with calbindin-D-28k antibody in Purkinje cells (arrow). Homogenisation changes of Purkinje cells. Magnification 200×.

Antibody staining for calbindin (CaBP, a calcium-binding protein) was performed on the cerebellum (Figure 9) of three of the studied cases. Thinning of the granular cell layers of the cerebral cortex was also observed (Figure 10). In comparison to the control group, in three cases (nos. 1, 2, and 5) there was weaker expression of this protein in Purkinje cells (Figure 10). Immunohistochemical staining showed normal expression of serotonin 5-HT_2A_ receptors in cortical neurons. Compared to the control group, the reaction seemed normal. Only one case showed no immunohistochemical reaction to this antibody (Table 3).

**Figure 11:**
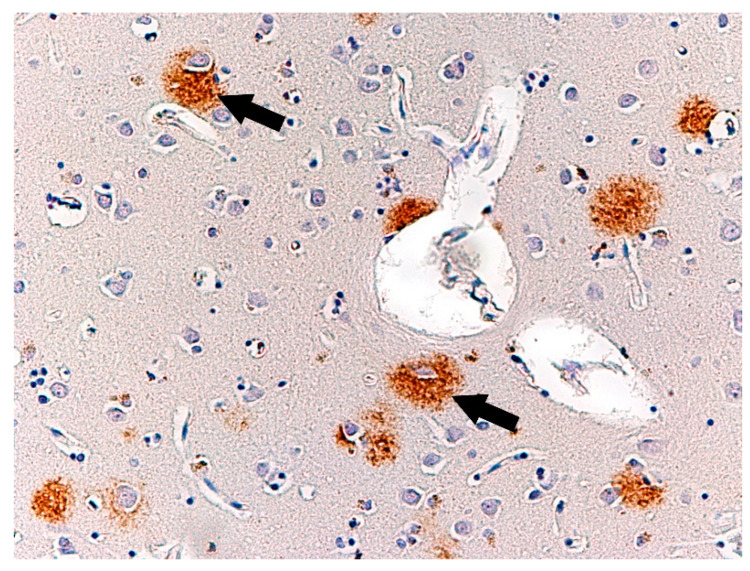
Case no. 2. Temporal lobe of the cortex. ß-amyloid_(1–42)_ deposits in senile plaques (arrows). Immunohistochemical reaction with ß-amyloid_(1–42)_ antibody. Magnification 200×.

Immunohistochemical reaction to β-amyloid, showed clusters of β-amyloid (1–42) isoform (Figure 11) in senile plaques of the occipital lobe cortex in three of the studied cases, while deposits of ß-amyloid (1–40) showed in only two cases (Table 3). We did not observe any pathological changes in the control group (Table 4). Furthermore, we performed immunohistochemical reactions with a-synuclein and ubiquitin, but the results were not characteristic.

## 4. Discussion

The results indicate the existence of neuropathological changes in the brains of the perpetrators of murder–suicide. The analysed material came from the frontal, parietal, occipital, and temporal lobes with the Brodmann area 28, basal ganglia, midbrain, and, in some cases, from the mammillary body and cerebellum. The study group was diverse in terms of age, education, social status, as well as the type of murder and suicide commit-ted. Nevertheless, in 50% of the histologically stained brain sections, there was evidence of neurodegenerative changes in the form of senile plaques in the cerebral cortex, as well as the presence of the initial stage of neurofibrillary degeneration.

Immunohistochemical reaction to ß-amyloid also revealed multiple deposits of this protein in the studied cortex sections in the form of senile/amyloid plaques and perivascular diffuse plaques. The changes described are found in the clinical picture of neurodegenerative diseases, such as Alzheimer’s disease, in which there is cognitive impairment. Importantly, the clinical data excluded information that the subjects had a family history of Alzheimer’s disease. The clinical picture of neurodegenerative diseases may include behavioural disorders, depressive symptoms, agitation, sometimes leading to aggression. Studies indicate that delusional disorders (Capgras syndrome) may accompany Alzheimer’s disease in up to 73% of cases, and hallucinations in nearly 49% of cases [17,18,19]. The formation of abnormal forms of ß-amyloid occurs almost throughout human life, but the mechanisms responsible for maintaining them at non-toxic levels are among the major factors involved in the pathogenesis of Alzheimer’s disease [20]. The presence of numerous senile plaques is observed in the brains of elderly people, over the age of 65. However, in our study these changes were observed in subjects at much younger ages of 33, 34, 58, and 60 years. In the literature, the link between impulse dyscontrol and atrophy patterns of AD has been described in several publications [21,22,23]. Our findings are in line with previous observations, supporting the influence of the process of atrophy in the crucial brain regions on behavior and impulse control.

Very important in the accumulation of abnormal peptide deposits seems to be the system responsible for removing metabolic waste products from the brain, which is de-void of lymphatic vessels. In the central nervous system, this is the role of the glymphatic system [24]. An important role in this waste clearance system, involving the transport of cerebrospinal fluid along with pathological substances, is played by astrocytes and the aquaporin 4 expressed by the processes in which they are involved [25]. In all the brain fragments studied, significant damage to the astrocytes, which mainly affected their processes, was also observed, suggesting a possible malfunction of the glymphatic system. In two cerebellums out of three studied, an attenuated immunohistochemical response to calbindin (CaBP, a calcium-binding protein) was observed [26]. Ca^2+^ is a unique ion that is involved in many important physiological processes, including the functioning of neurons and astrocytes [27,28]. Calcium ions regulate the release and synthesis of neurotransmitters, hormones, axonal transport, control of enzymatic re-actions, gene transcription, and many other processes throughout the course of human life, starting from the prenatal development period [29,30]. The findings of the study of this non-statistical amount of material are only an indication of future research goals. No significant changes in immunohistochemical expression compared to the control group were observed for the glutamate transporter of neuronal origin (GLAST) and the glutamate transporter of glial origin (GLT1), which does not exclude the involvement, especially with the observed chronic neuronal damage, of the existence of synaptic glutamate deficiencies leading—as in schizophrenia—to psychiatric disorders [31].

The normal expression of serotonin 5-HT2A in cortical neurons, in seven out of eight brains studied, may tentatively support the theory of researchers at the Baker Heart Re-search Institute in Melbourne (Australia) and the researchers at Wayne State University (USA), who maintain that depression or panic disorder (panic anxiety) can go hand in hand with an increase rather than a decrease in serotonin levels [32]. According to some researchers, there are certain groupings of serotonin neurons in the brain that are over-active in people suffering from depression [33]. Interestingly, features of increased impulsivity were observed in analyses of symptoms of depressive disorders [34]. Clinically, drugs affecting serotonergic transmission can be used in the prevention of impulsive and aggressive behavior [35,36]. In contrast, the generalised oedema and ischemic neuronal degeneration observed in the brain sections we studied should be associated with the direct cause of death in patients, complicated by brain ischaemia and/or hyperaemia and hypoxia.

Referring to the literature, the most frequent type of murder among suicide killers is the murder of a sexual partner with whom the perpetrator was in a relationship. Murderers are most often men who kill their female partners. It is almost unheard of for a woman to be the killer who takes her own life after a murder [37,38]. In the motivation process of women’s crimes against their partners, one can find relationships where, prior to murder, women remained in the role of deeply tormented victims embedded in the reality of an attitude of acceptance of violence, and thus the sense of guilt after the act was diminished to such an extent that it was not a factor pushing them to suicide [39,40]. In the literature, murder with subsequent suicide is also referred to as dyadic death or post-aggression suicide [41,42,43]. Descriptions of murder–suicide perpetrators usually present the perpetrator as a middle-aged man and often under the influence of alcohol during the act. The analysis of the perpetrator’s motivation processes most often focuses on the features of abnormal personality and the particular pressure of psychosocial stressors in the form of partnership conflicts, erotic jealousy, or financial difficulties [44,45,46,47,48]. Sometimes, especially when the homicide itself is exceptionally brutal, psychotic symptoms associated with exacerbation of mental illness or treatment interruption are identified in perpetrators [49]. Unfortunately, we cannot trace the motivational process and ascertain possible mental disorders in our study group because, due to the retrospective nature of the study protocol, we did not collect medical or behavioral data on the perpetrators. Undoubtedly, in subsequent studies such correlations and tracking of relationships would be extremely interesting.

The selection of a homogeneous sample group could contribute to establishing a more precise correlation between neuropathological changes in suicidal murderers and pathogenic factors that may influence behaviour at the time of such an act and possibly contribute to the selection of protective strategies in the future. Limitations of the current study are related to a lack of complete medical documentation and spare description of the violent behavior.

## 5. Conclusions

The presence of neurodegenerative changes at such a young age in some murderers may suggest preclinical lesions that affect cognitive functions and are associated with depressed moods.

## Figures and Tables

**Table 1:** Study group.

No.	Age	Sex	Tested Structure	Time of Autopsy after Death	Cause of Death
1	34	Male	Frontal lobe, cerebellum, basal ganglia	24 h	Hanging
2	58	Male	Frontal, occipital, and temporal lobes, basal ganglia, cerebellum	72 h	Exsanguination
3	54	Male	Frontal and temporal lobes with the Brodmann area 28	72 h	Fall from height
4	19	Male	Frontal, occipital, and temporal lobes, mammillary bodies	72 h	Hanging
5	60	Male	Frontal and temporal lobes with the Brodmann area 28, cerebellum	72 h	Hanging
6	34	Male	Frontal and temporal lobes, basal ganglia	24 h	Fall from height
7	33	Male	Frontal, occipital, and temporal lobes, thalamus	24 h	Exsanguination
8	37	Male	Frontal, occipital, and temporal lobes, basal ganglia, midbrain	72 h	Hanging

**Table 2:** Control group.

No.	Age	Sex	Tested Structure	Time of Autopsy after Death	Cause of Death
1	55	Male	Frontal, occipital, and temporal lobes, basal ganglia, cerebellum	96 h	Traffic accident
2	27	Male	Frontal, occipital, and temporal lobes, basal ganglia, cerebellum, cerebellum	96h	Traffic accident
3	39	Male	Frontal, occipital, and temporal lobes, basal ganglia, cerebellum	96 h	Traffic accident
4	21	Male	Frontal, occipital, and temporal lobes, basal ganglia, mammillary bodies, cerebellum	72 h	Traffic accident
5	29	Male	Frontal, occipital, and temporal lobes, basal ganglia, cerebellum	72 h	Traffic accident
6	42	Male	Frontal, occipital, and temporal lobes, basal ganglia, cerebellum	96 h	Traffic accident
7	80	Male	Frontal, occipital, and temporal lobes, basal ganglia, midbrain, cerebellum	72 h	Traffic accident
8	43	Male	Frontal, occipital, and temporal lobes, basal ganglia, midbrain, cerebellum	72 h	Traffic accident

**Table 3:** Histological and immunohistochemical findings. (+) immunohistochemical reaction present, (−) no immunohistochemical reaction, (×) no immunohistochemical staining performed.

No.	Age	Bielschowsky’s Stain	βA_(1–40)_	βA_(1–42)_	GFAP	Serotonin 5-HT_2A_	GLT1	GLAST	Calbindin-D28k
1	34	Senile plaques in the frontal lobe	−	+	+	+	+	+	+
2	58	Senile plaques in the occipital lobe	+	+	+	−	+	+	+
3	54	−	−	−	+	+	+	+	×
4	19	−	−	−	+	+	+	+	×
5	60	Senile plaques in the occipital lobe	−	+	+	+	+	+	+
6	34	−	−	−	+	+	−	−	×
7	33	Senile plaques in the frontal lobe	+	−	+	+	−	−	×
8	37	−	−	−	+	+	+	+	×

**Table 4:** Histological and immunohistochemical findings. (+) immunohistochemical reaction present, (−) no immunohistochemical reaction.

No.	Age	Bielschowsky’s Stain	βA_(1–40)_	βA_(1–42)_	GFAP	Serotonin 5-HT_2A_	GLT1	GLAST	Calbindin-D28k
1	55	−	−	−	+	+	+	+	+
2	27	−	−	−	+	+	+	+	+
3	39	−	−	−	+	+	+	+	+
4	21	−	−	−	+	+	+	+	+
5	29	−	−	−	+	+	+	+	+
6	42	−	−	−	+	+	+	+	+
7	80	−	−	−	+	+	+	+	+
8	43	−	−	−	+	+	+	+	+

## Data Availability

Not applicable.
